# Thrombophagocytosis by neutrophils as a spurious cause of thrombocytopenia

**DOI:** 10.1002/jha2.139

**Published:** 2021-05-05

**Authors:** Erica‐Mari Nell, Marthinus Johannes Dicks, Monalisa Ntobongwana

**Affiliations:** ^1^ Division of Haematopathology Department of Pathology Faculty of Medicine and Health Sciences Stellenbosch University Stellenbosch South Africa; ^2^ National Health Laboratory Services Tygerberg Hospital Cape Town South Africa

A 35‐year‐old pregnant woman presented with uncontrolled hypertension at 38 weeks gestation. A complete blood count was conducted as part of routine workup. Her haemoglobin was 9.5 g/L, with an absolute neutrophil count of 11.6 × 10^9^/L and platelet count measured at 42 × 10^9^/L by automated analyser. A peripheral blood smear showed a leucocytosis with neutrophil predominance. The majority of neutrophils (panels 1‐4) and the occasional monocyte (panel 1) contained large vacuoles with phagocytosed platelets (Figure 1). Platelet satellitism was also observed.

**FIGURE 1:**
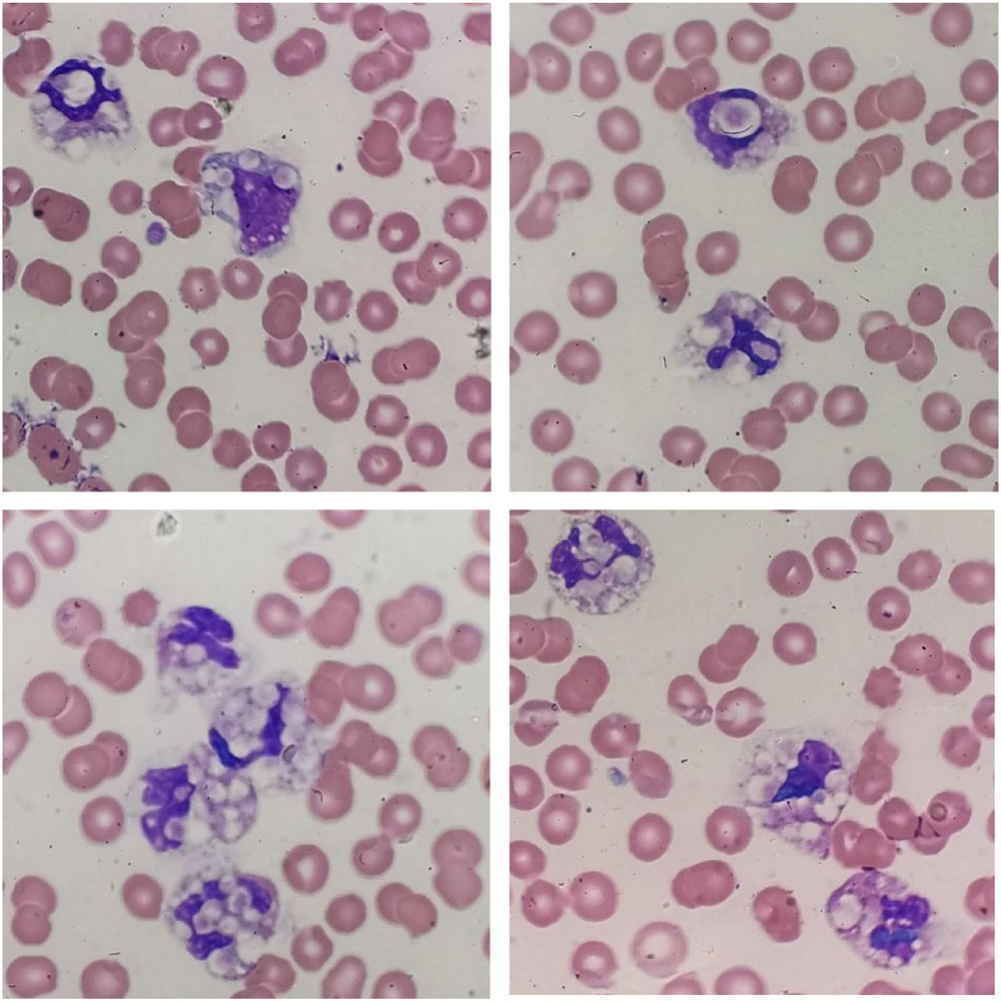
Micrograph showing platelet phagocytosis by neutrophils (all panels) and monocytes (upper left panel) as an in vitro phenomenon mediate by EDTA. Original magnification 100x. See supplementary file for video footage of this phenomenon.

Ethylenediaminetetraacetic acid (EDTA) dependent platelet phagocytosis is a rare cause of pseudothrombocytopenia. Other, more common causes of EDTA‐mediated pseudothrombocytopenia include platelet clumping and platelet satellitism. In order to confirm the in vitro nature of the observed platelet phagocytosis, a repeat specimen was collected in sodium citrate. This specimen showed a normal platelet count of 214 × 10^9^/L by automated analyzer and the absence of platelet phagocytosis on peripheral blood smear.

A wet preparation of whole blood taken from a K_2_‐EDTA tube and diluted with 0.9% saline was examined under microscopy and demonstrated the formation of neutrophil pseudopods around satellite platelets with subsequent platelet engulfment into cytoplasmic vacuoles (see Video).

In this patient, approximately 80% of platelets were phagocytosed. This is in keeping with previous case reports, such as in a healthy 22‐year‐old male in a phase I drug trial [2] and an 81‐year‐old woman with sepsis secondary to *Escherichia coli* pyelonephritis [3]. EDTA mediates this phenomenon by chelating and thereby sequestering the Ca^2+^ ions from the platelet membrane. The resulting conformational change of the platelet membrane leads to the exposure of cryptantigens on glycoprotein IIb/IIIa on circulating platelets. Autoantibodies can bind to these cryptantigens and trigger the EDTA‐mediated pseudothrombocytopenia [1]. The mechanism for auto‐antibody production remains unclear, as autoantibodies are not present when blood is anticoagulated with heparin or citrate.

In conclusion, platelet phagocytosis appears to be an extension of platelet satellitism which is dependent on EDTA exposure and autoantibodies binding to platelet cryptantigens. Awareness of these EDTA‐mediated phenomena can prevent unnecessary investigation, needless delays in management such as withholding medications or cancelling surgery, or the administration of unnecessary platelet transfusion for presumed thrombocytopenia.

## CONFLICT OF INTEREST

The authors declare that there is no conflict of interest.

## INFORMED CONSENT

Patient's consent was obtained for publication of the case.

## AUTHOR CONTRIBUTIONS

Erica‐Mari Nell and Marthinus Dicks were the primary investigators and partook in data and image collection and writing of the manuscript. Dr Monalisa Ntobongwana was the supervisor to the case report and assisted in critical appraisal of the work and contributed to the writing of the manuscript.

## Supporting information

Supporting Information

## Data Availability

All data generated during this study are included in this published article.
